# MicroRNA differential expression spectrum and microRNA-125a-5p inhibition of laryngeal cancer cell proliferation

**DOI:** 10.3892/etm.2021.9977

**Published:** 2021-03-24

**Authors:** Xiang-Dong Yao, Ping Li, Ji-Sheng Wang

Exp Ther Med 14:1699-1705, 2017; DOI: 10.3892/etm.2017.4685

An interested reader drew to our attention the fact that the data shown in Figs. 1-5 and in Table I in the above paper had already appeared in the following paper, published in the *Chinese Journal of Pathophysiology* in 2013 by a different research group [Zhang S-y, Lu Z-m, Song X-h, Zhang H-b, Chen L-s, Luo X-n, Chen S-h and Wu Y-l: Differential microRNA expression profile in laryngeal cancer and effect of miR-125a-5p on proliferation of laryngeal cancer cell line. Chin J Pathophysiol 29: 86-92, 2013], 2 years prior to its submission to *Experimental and Therapeutic Medicine*.

Following an investigation, the Editor of *Experimental and Therapeutic Medicine* was able to confirm that the data drawn to our attention were duplicated. This matter was taken up with the authors of the above article, who were unable to demonstrate that the data predated their appearance in *Chin J Pathophysiol*. On those grounds, the Editor of *Experimental and Theraputic Medicine* has decided that the above paper should be retracted. The Editor deeply regrets any inconvenience that this retraction has caused to the authors of the previously published paper, and to the readership of the Journal.

